# Recurrence Prevention in Patients with Urinary Tract Stone Disease

**DOI:** 10.1100/tsw.2004.3

**Published:** 2004-01-16

**Authors:** Hans-Göran Tiselius

**Affiliations:** Department of Urology, Karolinska University Hospital and Division of Urology, Center for Surgical Sciences, Karolinska Institutet, Stockholm, Sweden

**Keywords:** Calcium oxalate, calcium phosphate, cystine, diet, infection stone, medical treatment, recurrence prevention, residual fragments, uric acid

## Abstract

Formation of urinary tract concrements is a common disease and steps should be taken in order to elucidate the underlying mechanisms and to give the patients appropriate advice and medical treatment. This present article summarizes the principles for recurrence preventive measures in patients with uric acid, infection, cystine and calcium stone disease. Categories of stone formers are identified with the aim of providing a basis for an individualised treatment with a reasonable patient's compliance. The recommendations are in line with those given by the EAU guideline group for urolithiasis.

## INTRODUCTION

Formation of stones in the urinary tract is a common clinical problem. This disease has a prevalence of approximately 10–15%. The annual incidence has been estimated roughly to 1500–2000 in a population of 1 million. With a recurrence risk of around 50%, these patients constitute a considerable strain to the health care system[1,2,3,4,5].

Apart from the need for active stone removal in about 500 out of the 2000 patients, medical assistance is necessary for most of the remaining patients despite spontaneous passage of the stones.

To reduce the patients' suffering from the disease, the negative effects on renal function, and treatment costs, it is desirable to provide a recurrence preventive programme. The purpose of that is to arrest or at least reduce the rate of further stone formation.

There is no doubt that the interest in metabolic evaluation and identification of risk factors, as well as recurrence preventive measures, was decreased when noninvasive or low invasive methods were introduced for stone removal. Although the new technology undoubtedly was a dramatic breakthrough in the treatment of patients with stones, it needs to be emphasised that the recurrence rate persists or might in fact be increased.

One problem in the management of stone-forming patients is that there is no real consensus on how effective our current methods for stone prevention are. There are only a small number of randomised or controlled studies and in such, as well as other studies, the reported treatment periods are often too short to demonstrate convincingly the value of various metaphylactic measures. To these problems add low compliance[5,6,7,8]. Another complicating factor is that the mechanisms of stone formation are not understood satisfactorily.

Despite these shortcomings, it is essential to give the stone-forming patient medical advice and treatment in accordance with the demonstrated or predicted severity of the disease. This paper summarises the major considerations and therapeutic recommendations that presently should be made for these patients.

## CATEGORIES OF STONE FORMERS

Inasmuch as the treatment will be different for subgroups of patients, the first step of a biochemical work-up is an appropriate analysis of the stone composition[9,10,11].

Thereby we get useful information about whether the patient has formed a stone composed of calcium salts such as calcium oxalate and/or calcium phosphate or noncalcium salts such as uric acid/urate, infection, or cystine material (see Table 1). In case a stone analysis has not been carried out, the radiographic appearance of the stone, a sodium nitroprusside test for demonstration of cystinuria, a urine culture, and microscopic identification of cystine or struvite crystals are useful tools in the diagnostic process. In terms of calcium stones, it is of therapeutic interest to find those patients in whom the stone was composed of pure calcium phosphate, particularly brushite (calcium hydrogen phosphate).

**TABLE 1**
**Subgrouping of Patients According to the Stone Composition**

| | | **Salts** |
|---|---|---|
| **CA** | Calcium stones | Calcium oxalate |
| | | Calcium oxalate/calcium phosphate |
| | | Calcium phosphate |
| **UR** | Uric acid/urate stones | Uric acid |
| | | Ammonium urate |
| | | Sodium urate |
| **INF** | Infection stones | Magnesium ammonium phosphate |
| | | Carbonate apatite |
| **CY** | Cystine stones | Cystine |

It is generally considered that in patients who are referred to the subgroups UR, INF, and CY, the recurrence risk is high and powerful recurrence preventive measures are almost always motivated[7,12].

For subgroup CA, the course of the disease and the risk of recurrent stone formation covers a wide range of possibilities. It is therefore of practical value to make an attempt to categorise the patients according to the severity of the disease. In this respect, it might be useful to distinguish between first, single (S), and recurrent (R) stone formers. The recurrent stone formers can have a mild (m) or severe (s) disease. Moreover, the presence of residual stones or fragments might make sense as a potential nidus for new stone development. The different categories of patients in subgroup CA are shown in Table 2[6].

Irrespective of the previous history of stone formation, a few *specific risk factors* should refer the patient to category $R_s$. In this respect, the following *specific risk factors* need to be considered: medical disease, pharmacological treatment or anatomical abnormalities known to be associated with calcium stone formation, start of stone formation below the age of 25, stone composed of brushite, or a pronounced family history of stone formation.

**TABLE 2**
**Categories of Stone Formers in Subgroup CA**

| | |
|---|---|
| $S_o$, $R_{mo}$ | First (S) or recurrent stone former with a mild disease ($R_m$) and without residual stones or fragments (o). |
| $S_{res}$, $R_{mres}$ | First (S) or recurrent stone former with a mild disease ($R_m$) and with residual stones or fragments (res). |
| $R_s$ | Recurrent stone former with a severe disease or any calcium stone-forming patient with specific risk factors. |

It is a delicate matter to make a clear-cut distinction between categories $R_m$ and $R_s$. In the author's opinion, any patient who has formed at least four stones can be considered to have a severe disease, but it is important to include in this decision the patient's opinion and his or hers motivation for recurrence preventive measures.

## PRINCIPLES FOR PREVENTION OF RECURRENT STONE FORMATION

### Subgroup UR

Most patients in this subgroup have formed one or more stones composed of uric acid (or occasionally another urate). Since this uric acid precipitates in acid urine with or without an increased urinary concentration of urate, the basic treatment includes alkalisation and dilution of the urine. All these patients are recommended to increase their intake of fluids so that the 24-h urine volume is 2 l or more. Alkalisation can be accomplished with potassium citrate 7–10 mmol two to three times daily or sodium potassium citrate 7–9 mmol two to three times daily. In patients with a high serum urate or a high urinary urate, allopurinol should be added in a dose of 300 mg daily[12,13]. It needs to be emphasised that uric acid stones can be dissolved by oral chemolysis. The author thereby gives 7–10 mmol of potassium citrate three times daily and 300 mg of allopurinol together with recommendations of a high fluid intake.

Prevention of sodium urate and ammonium urate stone formation requires reduction of the urinary concentration of urate with allopurinol and in the case of sodium urate, a reduced salt intake also. Ammonium urate is commonly the result of a high concentration of urate and infection with urease-splitting organisms. In the latter case it is necessary to eradicate the infection[14,15].

There are, unfortunately, no chemolytic possibilities for stones companion ammonium urate or sodium urate.

### Subgroup INF

The successful long-term recurrence prevention of infection stones is based on an efficient removal of stone material from the kidney. Follow-up with administration of antibiotics during a period of 3–6 months after stone removal is considered necessary for eradication of the infection. It has been shown that sterilisation of urine can be accomplished also in the presence of small residual stone fragments.

Acidification of urine with methionine 500 mg three times daily or with ammonium chloride 1.5 g daily or 4.5 g one day a week might be useful to counteract precipitation of magnesium ammonium phosphate and carbonate apatite[16].

It is also important to keep in mind that some patients with infection stones have an underlying calcium stone disease that requires due attention[17].

## Subgroup CY

Less than 1% of stone formers suffers from the genetic disease cystinuria. The stone formation in these patients might be really aggressive and it is necessary to seriously consider preventive alternatives.

In order to reduce the concentration of urinary cystine, the fluid intake should be kept at a level that results in 24-h urine volumes exceeding 3 l. The solubility of cystine increases in alkaline urine and although the clinical effect of alkalinisation usually is marginal, this is the first pharmacological option particularly in patients with low cystine concentrations. Administration of potassium citrate 7–10 mmol two to three times daily is recommended. When these therapeutic measures prove insufficient or when the 24-h urinary cystine exceeds 3 mmol, cystine complexing agents should be added. The most popular agent for this purpose presently is α-mercaptopropionylglycin (tiopronin, Thiola) given in daily doses between 250 and 1500 mg. The dosage should be determined by the therapeutic response in terms of the level of supersaturation with cystine[18]. The solubility and formation products of cystine are $1 \times 10^{-20}$ and $1.3 \times 10^{-20}$ $(\text{mmol/l})^3$, respectively (see [19]).

The use of penicillamine is presently less common because of side effects. Other therapeutic agents with reported effects are ascorbic acid and captopril. Although the theoretical basis for the use of ascorbic acid is attractive, its clinical effect is probably small.

## Subgroup CA

Three therapeutic levels might be considered for patients with calcium stone disease: general drinking and dietary advice, specific drinking and dietary advice, and pharmacological treatment[5,7].

### *General Drinking and Dietary Advice*

A recommendation to aim at a 24-h urine volume of at least 2 l is good for all stone formers[8]. General dietary recommendations should be given carefully in order not to cause unnecessary restrictions. The basic elements of the dietary advice are to reduce intake of oxalate rich foodstuffs, to maintain the calcium intake at a level of 20–25 mmol/d, and to avoid excessive intake of animal proteins[7,8,12].

This general regimen would be sufficient for patients in categories $S_o$ and $R_{mo}$ and there is usually no need for an extensive metabolic evaluation or follow-up of these patients. It is, however, useful to exclude hyperparathyroidism by analysis of serum or plasma levels of calcium or ionised calcium[20,21,22].

### *Specific Drinking and Dietary Advice*

Patients in categories $S_{res}$, $R_{mres}$ and $R_s$ are all at a potential risk of stone growth or new stone formation. A urine analysis should be carried out to disclose abnormalities in urine composition. It is a reasonable first step to make an attempt to correct urine abnormalities so that the risk of forming urine that is critically supersaturated with calcium oxalate and calcium phosphate is decreased. The arbitrary levels for critical levels of the ion-activity products $AP_{CaOx}$ and $AP_{CaP}$ used by the author are $1.5 \times 10^{-8}$ $(\text{mol/l})^2$ and $50 \times 10^{-15}$ $(\text{mol/l})^2$, respectively[23]. From the estimates of $AP_{CaOx}$ and $AP_{CaP}$, the 24-h volume necessary to adequately reduce the supersaturation can be derived.

In case of *mild hyperoxaluria*, there are no pharmacological methods available and dietary restrictions are the way to go. There should be a reduced intake of foodstuffs rich in oxalate. Particular attention should be directed against oxalate intake between meals, for instance chocolate and nuts. The dietary measures that might be undertaken to correct other urinary abnormalities are extensively outlined elsewhere[7,24,25]. Suffice it here to state that advice to reduce the intake of calcium should not be given unless it is obvious that a high urinary excretion of calcium is caused by an excessive intake of dairy products.

A measure of the protein intake can be obtained by analysing urinary urea. The patient's attention should be directed to the possibility of an excessive intake of animal protein[26]. The aim thereby should be to reduce such dietary components to a level below 80–100 g/d.

Specific dietary and drinking advice should be considered as a first-line treatment in all $S_{res}$, $R_{mres}$, and $R_s$ patients. It needs to be observed, however, that pharmacological prevention always should be considered in case of failure with the principles presented above. For the patients in category $R_s$ as well as for those in categories $S_{res}$ and $R_{mres}$ in whom high levels of supersaturation with calcium oxalate or calcium phosphate are recorded, the indication to use pharmacological agents is strengthened.

### *Pharmacological Treatment*

The basic principle for all therapeutic measures is to reduce the supersaturation below the levels where crystal formation might occur. Several ways might lead to this goal, but the general idea is to be as selective as possible. There are studies that have shown good results when potassium citrate was given in an unselective way[27,28], but a selective treatment might be more efficient and more logical from a medical point of view[29]. The choice of a pharmacological agent therefore should be based on the analytical findings of the individual urine variables.

*Hypercalciuria* can be reduced by administration of thiazides. Hydrochlorothiazide 25–50 mg/d, bendroflumethiazide 2.5–5 mg/d, and trichlorthiazide 4 mg/d have proven efficient in reducing stone formation[30,31,32,33,34]. There are indications that supplements of magnesium together with thiazides might be useful[35] and potassium should always be given to avoid hypokalemia and hypocitraturia.

Orthophosphate also reduces urinary calcium. Due to gastrointestinal side effects and the required dosage (1 g three times daily), it is a less desirable alternative. Treatment with orthophosphate can be considered, however, for those hypercalciuric patients who do not tolerate thiazides[7,36,37].

A *low excretion of citrate* is best increased by administration of potassium citrate (6–7 mmol two to three times daily)[38,39].

A *low urinary magnesium* is an uncommon finding in calcium stone formers. In case of magnesium deficiency (low S-magnesium), administration of magnesium oxide or magnesium hydroxide should be given. To avoid a concomitant increased calcium excretion, the combined treatment with a thiazide is recommended.

In patients with *enteric hyperoxaluria*, caused by intestinal oxalate hyperabsorption, the following therapeutic steps are recommended: restricted intake of oxalate, administration of calcium 0.5–1 g three times daily together with meals and potassium citrate 7–10 mmol two to three times daily, also taken with meals.

Allopurinol might be useful for patients with *hyperuricosuric calcium oxalate stone disease*. In these patients a daily dose of 300 mg allopurinol should be given. This type of stone formation is subject, however, to a considerable geographical variation[40,41].

For patients with or without a high supersaturation with calcium oxalate or calcium phosphate and without any specific abnormality in urine composition, potassium citrate or potassium magnesium citrate might be useful to augment the inhibition of crystal growth and crystal aggregation[42].

*Brushite* stones are rarely encountered and studies on their prevention are scanty. The need for powerful measures is obvious, however, in view of the high recurrence rate seen with this type of stone. The combined use of thiazide, magnesium, and potassium citrate is a tentative alternative. Even if the stone formation is not arrested, an increased magnesium/calcium ratio favourably counteracts the conversion of amorphous calcium phosphate to brushite[19].

## CONCLUSION

With careful identification of risk factors and appropriate steps to reduce the risk of forming urine that is critically supersaturated with the salts of relevance to stone formation, a reasonable control of the stone disease can usually be obtained. In terms of calcium stone formation, the ideal stone preventive programme is not yet available and increased knowledge of the stone-forming process is essential. Therefore stone formation cannot be arrested in all patients.

One important prerequisite to a successful outcome is that the patients are carefully followed and the urine composition monitored. The problem with poor patient's compliance cannot be overestimated.

## ACKNOWLEDGEMENT

The expert secretarial assistance by Ms. Marie Karlsson is highly appreciated.
